# Structural modifications and kinetic effects of *KRAS* interactions with *HRAS* and *NRAS*: an *in silico* comparative analysis of *KRAS* mutants

**DOI:** 10.3389/fmolb.2024.1436976

**Published:** 2024-08-09

**Authors:** Isaac Silverman, Michael Gerber, Aaron Shaykevich, Yitzchak Stein, Alexander Siegman, Sanjay Goel, Radhashree Maitra

**Affiliations:** ^1^ Department of Biology, Yeshiva University, New York, NY, United States; ^2^ Department of Oncology, Rutgers Cancer Institute of New Jersey, New Brunswick, NJ, United States

**Keywords:** *KRAS*, *HRAS*, *NRAS*, molecular dynamics simulation, mutation, proteomics

## Abstract

The RAS genes which code for *KRAS*, *HRAS*, and *NRAS* are three of the most frequently mutated oncogenes responsible for cancer deaths. Tumorigenesis is one of the most significant outcomes of deregulation of RAS GTPases. Although the structures have been extensively studied, there is still more to be discovered about the actual binding conformations of the three isoforms, especially when mutated, to design an inhibitory drug. Recent studies have identified important interactions between the three isoforms that affect the oncogenic strength of the others when they are mutated. In this study, we utilize molecular dynamics simulations to examine the modifications of the structural property, mechanism, and kinetic energy of *KRAS* when interacting individually and with *HRAS* and *NRAS*. Notably, we found that WT-*KRAS*’ orientation when bound to WT-*HRAS* vs. WT-*NRAS* is rotated 180°, with mutants demonstrating a similar binding pattern. The binding sites of the isoforms with *KRAS* share similarities with those involved in the GDP/GTP active site and site of *KRAS* dimerization. Thus, the isoform interaction can serve as an inhibitory method of *KRAS* actions. This study advances the understanding of inhibiting RAS-driven cancers through a novel isoform interaction approach only recently discovered, which has been proven to be an effective alternate therapeutic approach. We developed a blueprint of the interaction which would be beneficial in the development of *KRAS* mutant-specific and pan*-KRAS* mutant inhibitory drugs that mimic the isoform interactions. Our results support the direct interaction inhibition mechanism of mutant *KRAS* when bound to WT-*HRAS* and WT-*NRAS* by the isoforms’ hypervariable region binding to the G-domain of *KRAS*. Furthermore, our results support the approach of reducing the effects of oncogenic *KRAS* by altering the concentration of the isoforms or a drug alternative based on the overall structural and kinetic stability, as well as the binding strength of the mutant-isoform complexes.

## 1 Introduction

### 1.1 RAS proteins in cancer biology

Mutations in the rat sarcoma viral (RAS) oncogene family, specifically *KRAS, HRAS*, and *NRAS*, are among the most prevalent among human tumorigenesis found in 30% of all cancer types included. Most prominently, *KRAS* is mutated in 90% of pancreatic cancers, 45% of colorectal cancer, and 30% of lung cancer cases (Saperstein et al., 2023; Shaykevich et al., 2023).

Of the oncogenic mutations in the RAS family, the *KRAS* locus is the most often affected with a relatively higher prevalence in about 30% of cancerous tumors, while the *HRAS* and *NRAS* locus mutations are present in a modest 8% and 3%, respectively (Fernandez-Medarde and Santos, 2011). Each *RAS* mutation has been observed to predominately promote tumors in distinct cancer types. The highest levels of *KRAS* mutation have been observed in pancreatic carcinoma, colorectal cancer, and lung malignancies. *HRAS* protein mutations are more prone to appear in dermatological and head and neck cancers. *NRAS* protein mutations are most prevalent in melanomas and hematopoietic malignancies (Muñoz-Maldonado et al., 2019). Understanding the interactions will provide valuable insights into drug development that is much needed in cancer treatment.

The RAS proteins are considered binary molecular switches that cycle between activated and inactivated states when GTP and GDP are bound to them, respectively (Zinatizadeh et al., 2019). Within the cell, the conversion between the two states is regulated by guanine nucleotide exchange factors (GEFs) and GTPase activating proteins (GAPs). GEFs steer the replacement of GDP with GTP, thereby activating the RAS protein, while GAPs enhance GTP hydrolysis, effectively inactivating the RAS protein (Vigil et al., 2010). Most somatic mutations of RAS directly influence their binding competencies with GTP and compromise their ability to hydrolyze into the inactive state (Mukhopadhyay et al., 2021). Specifically, mutations at residues G12, G13, and Q61 introduce steric hindrance within the RAS protein structure, which prevents the effective binding of GAPs. Thus, the RAS proteins are effectively fixed in their active conformations (Scheffzek et al., 1997). Consequently, the overabundance of persistently activated RAS proteins over-induces numerous downstream transduction pathways, which are RAS-dependent, frequently resulting in tumorigenesis (Murugan et al., 2019). Noteworthy, RAS canonical downstream pathways include the mitogen-activated protein (MAP) kinase (RAS, RAF, MEK, and ERK) pathway and PI3K (PI3K, AKT, mTOR, and PTEN) pathway (Sapir et al., 2021).

It should be emphasized that the entire coding sequence structure of the GDP-/GTP-binding region and other effector binding sites is significantly similar for each of the three isoforms of RAS proteins (Prior et al., 2012). Despite the evidentiary redundant functionality, there are abundant data that indicate that each isoform also exhibits unique functions within the cell, specifically with regards to signaling (Omerovic et al., 2007). These distinctive roles can be attributed to the C-terminal hypervariable region of the RAS proteins (Henis et al., 2009). The focus of this paper is the G-domain (residues 1–166) of *KRAS*, in which the isoforms share >95% of the sequence structure, and its interaction with the C-terminal hypervariable regions of WT-*HRAS* and WT-*NRAS* (Hancock and Parton, 2005).

### 1.2 Significance of *KRAS* mutations in the development of RAS-driven cancer therapies

Recent large-scale analysis of tumor samples has notably revealed that the mutation rate differs for each of the three RAS isoforms within the G-domain. Although 99.2% of mutations occur at residues G12, G13, and Q61, the percentage of mutations at a specific residue greatly varies for each RAS isoform. Approximately 80% of *KRAS* mutations occur at residue G12, ∼15% at G13, and ∼5% at Q61. *HRAS* is similar for the fact that a majority of its mutations, ∼50%, occur at residue G12; however, the second largest percentage of its mutations, ∼40%, occur at Q61. Only ∼10% of its mutations occur at G13. In the most stark contrast with *KRAS*, the majority of *NRAS* mutations, ∼60%, occur at residue Q61, followed by ∼35% at G12 and ∼5% at G13 (Prior et al., 2012). Additional mutations at residues G18, V117, and L146 have also been observed in the RAS isoforms, although in insignificant amounts (Stolze et al., 2015). In *KRAS*, the most frequent mutations at the G12 residue are G12D, G12V, G12C, G12A, G12R, and G12S. At the G13 residue in *KRAS*, the most common mutation is G13D. Finally, in *KRAS*, the most prevalent mutation at the Q61 residue is Q61H (Muñoz-Maldonado et al., 2019). Of significance, residue G12 is located at the active site of the RAS proteins, which includes a p-loop at residues 10–17 and two switch regions (Switch I at residues 25–40 and Switch II at residues 60–74) (Gerber et al., 2022).

Different types of *KRAS* mutations are found disproportionally in cancer types dependent on internal and external factors involved in oncogenesis. For example, the G12C mutation is more common in lung cancer patients due to transversions of G:C > T:A which are correlated with the formation of bulky DNA adducts created by tobacco smoke mutagens (Prior et al., 2020). The G12A and G12V mutants in lung adenocarcinoma are also largely attributed to tobacco smoke, while the G12D mutation has been designated as a “clock-like” mutation (Cook et al., 2021). In a clinical study, the G12D mutation was noted to be the most prevalent *KRAS* mutant transgressor amongst pancreatic cancer patients, comprising 44.9% (115/256), and CRC patients, comprising 30.8% (45/146). However, in non-small-lung cancer, the G12C mutation is the most common with 35.9% (79/220) of patients possessing the mutation, with G12D as the second most frequent in 16.4% (36/220) of patients. This study concluded that patients harboring the G12D mutation had distinctive clinical and genomic characteristics compared with those with other mutation types (Lin et al., 2023).

The type of *KRAS* mutation observed in a patient often determines the therapeutic treatment used and disease prognosis. *KRAS* mutant inhibitors have been proven to be an effective treatment alone and used in combination therapies. Certain *KRAS* mutants, which are more prominent, such as G12C, have had more extensive progress in the development of stand-alone inhibitors and ones that can be coupled in dual-therapy treatments. These have been successful in treating patients affected by the G12C mutant in numerous cancer types, leading to more positive disease prognosis (Qunaj et al., 2023; Wu et al., 2023). One study of patients with G13D-mutated tumors treated with cetuximab for chemotherapy-refractory colorectal cancer had better prognosis outcomes than patients with the other mutations (Rabara et al., 2019). Progress has also been made in generating small-molecule inhibitors for *KRAS* mutants as well, each impacting the proteins’ tumorigenesis effect in unique ways (Shetu and Bandyopadhyay, 2022). Efforts have also been made to produce pan-*KRAS* inhibitors to offer a broader range of therapeutic effectiveness (Corcoran, 2023; Kim et al., 2023). This study aims to guide the drug design of mutant-specific and pan-*KRAS* inhibitors by utilizing RAS isoform interactions, which have proven to have suppressive effects on mutant *KRAS*-mediated oncogenesis.

### 1.3 Interactions with RAS isoforms suppress tumorigenic actions of mutant KRAS

A recent *in vivo* study has shown that when WT-*HRAS* and WT-*NRAS* interact with mutated *KRAS,* specifically G12D in lung cancer, it effectively reduces *KRAS* dimerization, as well as decreases the amount of ERK downstream signaling. These results support the claim that interactions between WT-*HRAS* and WT-*NRAS* with oncogenic *KRAS* reduce the tumorigenic effects of mutations in *KRAS* (Tang et al., 2023). This novel study indicated a potential inhibitory method of mutant *KRAS* through RAS isoform interactions, although the authors noted that additional investigation is needed to understand the mechanism through which WT-*HRAS* and WT-*NRAS* function to suppress mutant *KRAS*. Our study investigated a direct interaction between the isoforms as the mechanism responsible for the suppression of *KRAS* actions. Utilizing molecular dynamics simulations, we analyzed the effects of each *KRAS* mutation on the structural conformation and kinetic energy of each complex’s protein–protein interaction.

Numerous other studies listed on the STRING database support the claim that the three RAS isoforms interact with each other in several cancer types (Markowitz et al., 2016). According to the STRING database, the interaction between *KRAS* and *HRAS* has an experimental and biochemical data confidence interval of 0.893. The interaction between *KRAS* and *NRAS* has an experimental and biochemical data confidence interval of 0.847. The interaction between *HRAS* and *NRAS* has an experimental and biochemical data confidence interval of 0.877 (Szklarczyk et al., 2023).

### 1.4 An *in silico* methodology to understand the binding interactions between the RAS isoforms

Bioinformatic methods have proven to serve as an advantageous complement to traditional laboratory techniques, often establishing a foundation for experimental design *in vitro* and *in vivo*. Molecular dynamics simulations provide an in-depth understanding of the intricacies of genomics and proteomics, especially within the scope of the discovery of binding sites and interaction mechanisms between macromolecules (George Priya Doss et al., 2008). Additionally, extensive study is conducted to simulate the effects of drug treatments and their biological responses (Drusbosky et al., 2017; Sharma et al., 2020). Utilizing various computational analysis tools, including structural and energetic calculation software, the potential binding sites and interaction mechanisms can be explored. It is also possible to analyze interactions at a variety of temperatures, pressures, and timescales to determine the effects of binding between molecules (Huang et al., 2017). In studies attempting to analyze potential protein inhibitors, molecular dynamics simulations provide a plethora of information beneficial toward therapeutic developments (Saini et al., 2019; Salo-Ahen et al., 2020; Singh et al., 2022a; Singh et al., 2022b; Gao et al., 2023). Bioinformatic techniques also allow for the opportunity to investigate the effects of mutations on binding sites, interaction energy and mechanisms, and structural conformation of macromolecules (Kumar et al., 2013; Bhaumik et al., 2014; Gerber et al., 2022).

**FIGURE 1:**
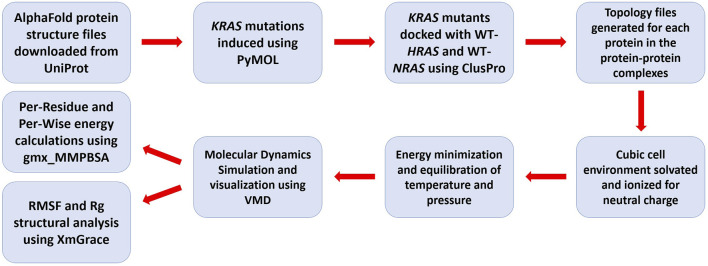
Flowchart of the molecular dynamics simulation methodology used in analyzing isoform interactions.

Utilizing the aforementioned techniques, our goal was to examine and understand how *KRAS* mutated at the G12, G13, and Q61 residues interacts with wildtype *HRAS* and *NRAS* in order to quantify and visualize the effects of the intra-familial interactions (Figure 1). Specifically, we aimed to demonstrate the initial binding interactions between the isoforms. We computationally modified an existing AlphaFold AI-predicted crystal structure of *KRAS* from the UniProt database to introduce the most frequent mutations at the G12, G13, and Q61 residues. Each mutant version of *KRAS* was individually docked with wildtype *HRAS*, a molecular dynamic simulation was performed, and structural modifications and kinetic effects were quantified and visualized. The same procedure was completed using the mutant versions of *KRAS* with wildtype *NRAS*. We performed a comparative analysis of each mutated simulation with a simulation between WT-*KRAS* and the respective RAS isoform. We proposed a direct inhibition mechanism for the suppressive effect of WT-*HRAS* and WT-*NRAS* and developed a structural and kinetic blueprint of the interactions. Using the blueprint, the specific residues that exhibited notable activity could be of importance for designing drugs that would mimic the interactions of WT-*HRAS* and WT-*NRAS* and have a similar effect. Additionally, the unique stabilities of the mutants and WT-*HRAS* and WT-*NRAS* would be beneficial in determining the concentration of WT-*HRAS* and WT-*NRAS* or a drug alternative that would be necessary to be effective.

## 2 Methods

### 2.1 Preparation and modification of *KRAS*, *HRAS*, and *NRAS* crystal structures

The AlphaFold crystal structures for human *KRAS* (ID: AF-P01116-F1), human *HRAS* (ID: AF-P01112-F1), and human *NRAS* (ID: AF-P01111-F1) were downloaded in a Protein Data Bank (PDB) format from the UniProt database and separately uploaded to PyMOL in individual sessions. In PyMOL, each protein structure was visualized in a three-dimensional (3D) form (Yuan et al., 2017). Each protein file contained a single protein chain (Chain A), with their respective 189 amino acid sequences. Chain A for both *HRAS* and *NRAS* was renamed “Chain B” to serve as a “ligand” when bound to Chain A of *KRAS*, functioning as a “receptor.”

### 2.2 Inducing mutation into the *KRAS* crystal structure

Nine copies of the modified *KRAS* crystal structure were created. One copy did not receive any mutations as it was designated as the wildtype copy. Each of the other eight copies was individually mutated in PyMOL at the G12, G13, or Q61 residues (Yuan et al., 2017). At the G12 residue, the glycine was replaced with one of the following amino acids: aspartic acid, cysteine, valine, alanine, arginine, and serine (G12D, G12C, G12V, G12A, G12R, and G12S). At the G13 residue, glycine was replaced with aspartic acid (G13D). At the Q61 residue, glutamine was replaced with histidine (Q61H).

### 2.3 Protein–protein docking

Each mutant *KRAS* crystal structure file and each wildtype *KRAS*, *HRAS*, and *NRAS* crystal structures were individually uploaded to PMV software (Sanner, 1999). Hydrogen atoms were added to polar regions, and Kollman charges were added, which gave the template values for each amino acid. On the ClusPro server, each version of mutant *KRAS* was uploaded as the receptor and WT-*HRAS* was uploaded as the ligand (Kozakov et al., 2017). The same was then performed for each version of mutant *KRAS* with WT-*NRAS*. WT-*KRAS* was also docked with WT-*HRAS* and then separately with WT-*NRAS*.

### 2.4 Protein complex topology preparation

Using GROMACS software, the simulated environment was prepared for the dockings of WT-*KRAS* and mutants paired with either WT-*HRAS* or WT-*NRAS* (Abraham et al., 2015). A 10-ns simulation was then run for each complex. Utilizing VMD software, the simulations were able to be visualized in video format to observe the protein–protein interactions for each complex (Humphrey et al., 1996). Structural, topology, and parameter files generated in the previous three steps are available at https://github.com/IVSilverman/RAS_Sim.

### 2.5 Protein structure analysis

To analyze the structure of each simulation, root mean squared fluctuation (RMSF) and radius of gyration (Rg) were plotted using XmGrace software (Turner, 2005). The RMSF is a calculation of the average displacement of each *KRAS* variant residue over the course of the 1,000 frame (10 ns simulation) while interacting with WT-*HRAS* or WT-*NRAS*. From this, the flexibility of specific segments of *KRAS* variant residues is able to be noted for interacting with WT-*HRAS* and WT-*NRAS*, respectively. The overall compactness of the protein structure and its flexibility is visualized using the RMSF graphs (Kleinjung and Martínez, 2015). The RMSF plots significantly indicate the regions of the proteins which are most mobile during the simulation. Often, increased mobility is associated with interaction activity either with another protein or ligand or itself. In complement with an analysis of complex energy, RMSF improves confidence that a specific region is active in interactions. RMSF also indicates regions which are mobile as a result of interactions at other sites. Rg calculates the root mean square average of the distance of all the proteins’ atoms from their centers of mass at each frame of the simulation, thus providing information about the structural stability (Sneha and George Priya Doss, 2016).

### 2.6 Protein energy analysis

gmx_MMPBSA software was utilized to calculate the interaction energy between the *KRAS* variants, and WT-*HRAS* and WT-*NRAS*, respectively, indicated contributing residues of the interaction (Baker et al., 2001a; Miller et al., 2012a; Kumari et al., 2014a). These residues contributed greater than 0.0100 kcal/mol of energy to the complex.

In the MMGBSA (Generalized Born model) (Genheden and Ryde, 2015) output, four energy values of the contributing residues were produced. First, the energy values for the bound state of all the contributing residues were available as the total complex energy. These values were used as a measure of stability. Additionally, the energy values for the unbound states of each protein were available as the receptor and ligand energies. In all simulations conducted, WT-*HRAS* and WT-*NRAS* were set as the receptors, although this did not have any effect on the results. Finally, the delta energy was available, which indicated whether the interaction between the proteins was thermodynamically favorable. Delta energy (*interaction energy*) = Total Complex (*bound state*) - [Receptor + Ligand] (*unbound states*). Entropy was not calculated, so the delta energy was equal to the change in enthalpy (𝚫H). Of most interest were the total complex energy graphs to understand the effect of the mutations on the stability of the complexes and the delta energy graphs to determine the impact of the mutations on the binding strength of the proteins (Miller et al., 2012b).

The amount of contributing residues was condensed in the delta graphs because not all contributing residues fluctuate significantly in energy between bound and unbound states. Only residues that do were included in the delta graphs. These were specifically of interest as the points of interaction between the proteins.

Two analysis modes of gmx_MMPBSA software were used: per-residue and per-wise. Utilizing the per-residue analysis of gmx_MMPBSA software, the total energy of the contributing residues with all other contributing residues of both proteins (the *KRAS* variant and *HRAS* or *NRAS*, respectively) was calculated. This was analyzed as residue–complex interaction energies. Utilizing the per-wise analysis of gmx_MMPBSA software, specific residue–residue interactions were calculated. This analysis further broke down the residue–complex energies into more individual interactions. In per-residue analysis, it could only be noted that a specific residue had a total energy of interaction with the entire complex. In per-wise analysis, that total energy was separated into the energies of distinct residue–residue interactions. Thus, the sum of all the per-wise energies for a specific residue would be equal to the per-residue energy for that residue. This was analyzed as residue–residue interaction energies.

gmx_MMPBSA software produced four figure types for the entire complex for each type of analysis: line plot, bar plot, PyMOL visualization, and heatmap plot.

The line plot represented the total sum of all the contributing residues per frame of the simulation. The bar plot represented the average energy of each contributing residue throughout the simulation. PyMOL visualization could also be created that projected the bar plot data onto 3D structures of the proteins. The heatmap results differ between the per-residue and per-wise heatmap plots. The per-residue heatmap plot depicted the amount of energy of each contributing residue per frame of the simulation. The per-wise heatmap plot depicted the contributing residue–residue interaction energies (Valdés-Tresanco et al., 2021).

In addition to these figures for the entire complex, variations in them are available for specific residues in each analysis mode. This is particularly useful to determine the type of bonding between specific residues in per-residue and to analyze the residue–residue interactions per-wise. These figures were available for the total complex, receptor, ligand, and delta energies.

### 2.7 Software

#### 2.7.1 ClusPro

The ClusPro server is a tool used for protein–protein docking. The server inputs two files in Protein Data Bank (PDB) format and produces a number of docked conformations structured according to different advanced parameters (Kozakov et al., 2017).

#### 2.7.2 Python Molecule Viewer

Python Molecule Viewer (PMV) software is used to edit protein structure and manipulate its environment by altering charge and solvation. It also produces 3D visualizations of the protein structure (Sanner, 1999).

#### 2.7.3 PyMOL

PyMOL is a program capable of editing protein sequences to induce mutations and developing 3D visualizations of the protein structure. The PyMOL Molecular Graphics System, Version 2.0 Schrodinger, LLC was used (Yuan et al., 2017).

#### 2.7.4 GROMACS

GROMACS is a Linux-based molecular dynamics simulation software that renders a topology regulated by Newton’s laws that can be employed to generate interactions between biomolecules *in silico*, such as proteins (Abraham et al., 2015). GROMACS Version 2020 was used (Lindahl, 2020).

#### 2.7.5 gmx_MMPBSA

gmx_MMPBSA is a powerful python program designed to derive energy calculations from molecular dynamics simulations. Version: gmx_MMPBSA v1.5.1 (Baker et al., 2001b; Miller et al., 2012b; Kumari et al., 2014b).

#### 2.7.6 XmGrace

XmGrace is a plotting software application capable of generating 2D graphs of data files (Turner, 2005).

### 2.8 Visual Molecular Dynamics

Visual Molecular Dynamics (VMD) is a software application utilized to visualize results of molecular dynamics simulations (Humphrey et al., 1996). Simulation files are processed into molecular models and complex interactions are able to be observed in video format. The two simulation files input into VMD were the post-simulation files with extensions .gro. and .tpr.

## 3 Results

### 3.1 *KRAS* mutation torsional strain

When generating a missense mutation in *KRAS*, torsional strain was produced in the protein. Torsional strain occurs due to unfavorable steric interactions in the new conformation between the atoms of the mutated residue and the other residues. Multiple conformations (rotamers) were possible when the mutation was induced. The rotamer of each variant with the least torsional strain was chosen for simulation to allow for the greatest bond flexibility. In v *KRAS*, no torsional strain was measured by PyMOL. All variants except G12A, due to its small size similar to glycine, resulted in an increased level of bond restriction. G12V had the highest torsional strain (Table 1).

**TABLE 1:** Torsional strain of *KRAS* variants.

*KRAS* variant	Induced torsional strain
Wildtype	—
G12D	35.55
G12C	39.2
G12V	56.87
G12A	—
G12R	23.61
G12S	29.57
G13D	9.65
Q61H	13.29

Torsional strain values of each *KRAS* variant were calculated by PyMOL, when the mutations were induced and are listed. Wildtype *KRAS* did not exhibit any torsional strain in its native conformation. The G12A variant did not exhibit multiple rotamers or any torsional strain after mutation.

### 3.2 Structural modifications

#### 3.2.1 Qualifying the stability of interaction by residue: RMSF fluctuations

##### 3.2.1.1 HRAS

**FIGURE 2:**
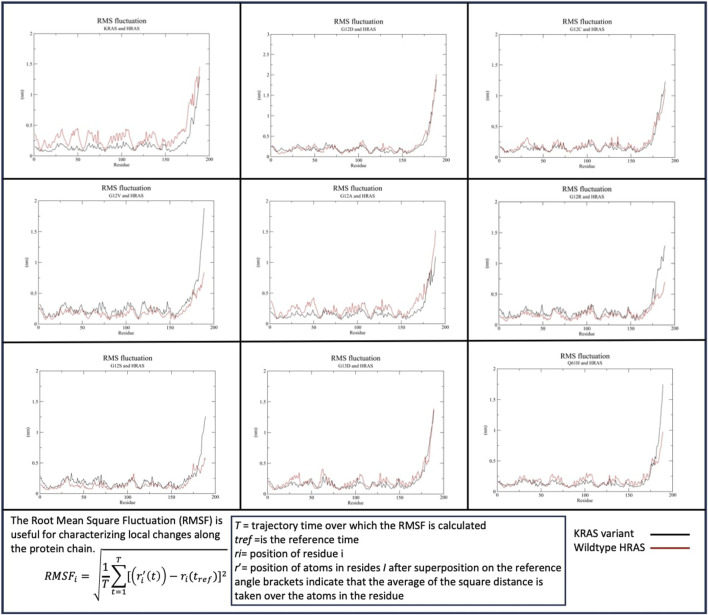
Root mean squared fluctuation (RMSF) of the *KRAS* variants with WT-*HRAS*. The average fluctuation of each residue in each *KRAS* variant and WT-*HRAS* are plotted. The regions of highest mobility are the c-termini of both proteins. *KRAS* mutation was shown to affect the c-termini mobility the most and decreased the fluctuation of WT-*HRAS*.

RMSF data indicated c-terminus residues in the *KRAS* variants and in WT-*HRAS* and WT-*NRAS* were the most mobile in their interactions (Figure 2). When WT-*KRAS* interacts with WT-*HRAS*, the c-terminus residues of both proteins fluctuate at ∼1.5 nm G12D, G12C, and G13D all displayed similar fluctuations as WT-*HRAS* in their c-termini, similar to WT-*KRAS*. However, when WT-*HRAS* interacts with G12R, G12S, G12V, and Q61H, its fluctuation notably decreased. Notably, G12V and Q61H were the only mutants to increase their fluctuation to ∼1.75–2 nm when interacting with WT-*HRAS*. G12A had the largest decrease in fluctuation of all the mutants to ∼1.1 nm. The fluctuation of the rest of both protein bodies was relatively stable among all mutants and WT-*HRAS*, remaining below 0.5 nm throughout the simulations. Interestingly, the body of WT-*HRAS* had the most fluctuation with WT-*KRAS* while remaining more equivalent to the fluctuation with the *KRAS* mutant in other simulations. G12A was the only one to display similar behavior.

##### 3.2.1.2 NRAS

**FIGURE 3:**
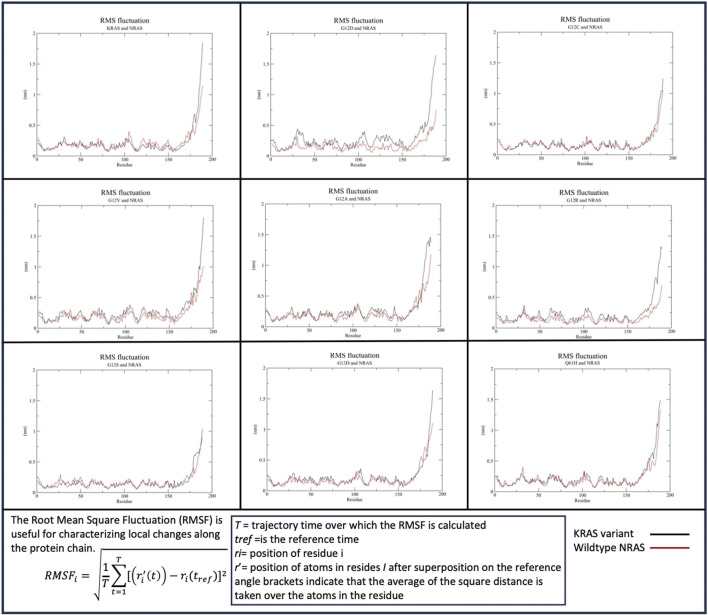
Root mean squared fluctuation (RMSF) of the *KRAS* variants with WT-*NRAS*. The average fluctuation of each residue in each *KRAS* variant and WT-*HRAS* are plotted. The regions of highest mobility are the c-termini of both proteins. *KRAS* mutation was observed to affect the c-termini mobility the most and decreased the fluctuation of WT-*NRAS*.

Similar to *KRAS* and *HRAS* interactions, the c-termini of the *KRAS* variants and WT-*NRAS* were both most active when binding (Figure 3). A significant difference between WT-*HRAS* vs. WT-*NRAS* interactions with *KRAS* was that the c-terminus of WT-*KRAS* and most mutants with *NRAS* consistently displayed greater fluctuation than the c-terminus of WT-*NRAS*. The c-terminus of WT-*KRAS* fluctuated to ∼1.6–2 nm with WT-*NRAS*, which fluctuated at ∼1–1.25 nm G12V and G13D had the most similar fluctuation patterns as the wildtype simulation. G12D and G12R had decreases in fluctuation of both proteins. G12A distinctly displayed a wavy maximum fluctuation at a similar fluctuation level as WT-*KRAS*. Notably, in the G12C, G12S, and Q61H mutant complexes, the fluctuation of the c-termini of both proteins became more equivalent to each other. G12C and WT-*NRAS* involved a decrease in G12C fluctuation. G12S and WT-*HRAS* involved a decrease in both G12C and WT-*HRAS* fluctuations. Q61H and WT-*NRAS* involved a decrease in Q61H fluctuation and an increase in WT-*NRAS* fluctuation. The fluctuation of the rest of both protein bodies was relatively stable among all mutants and WT-*NRAS*, remaining below 0.5 nm throughout the simulations. All WT-*KRAS* and mutant bodies had near-equivalent fluctuation values with WT-*HRAS*, with the exception of G12D, which had slightly more fluctuation in the mutant.

#### 3.2.2 Protein shape analysis: radius of gyration

##### 3.2.2.1 HRAS

**FIGURE 4:**
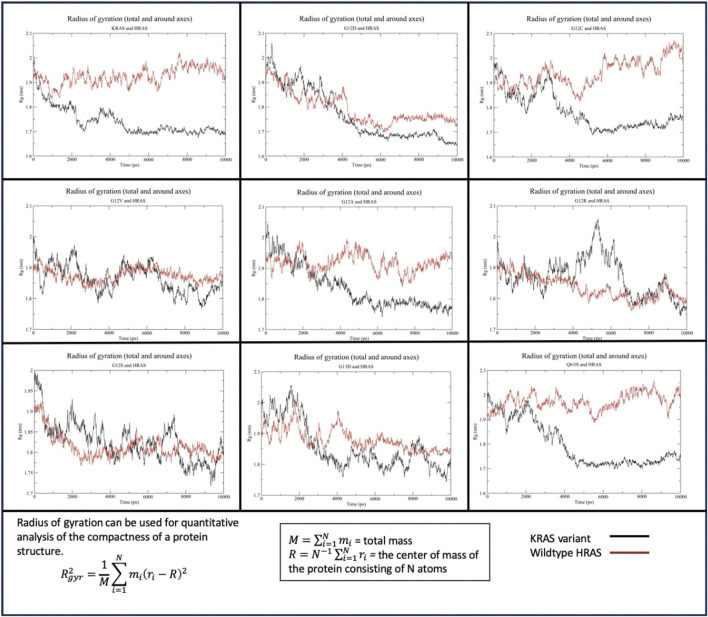
Radius of gyration (Rg) of the *KRAS* variants with WT-*HRAS*. The average distance protein residues from the center of the protein were plotted against time for the *KRAS* variants and WT-*HRAS*. A smaller Rg signified a denser protein, while a higher Rg indicated a decrease in overall compactness. Mutation in *KRAS* was observed to influence the density of both proteins.

The Rg of WT-*KRAS* interacting with WT-*HRAS* indicated that WT-*KRAS* increased in density throughout the simulation before equilibrating at ∼1.7 nm, compared to WT-*HRAS* which remained at a more consistent density of 1.9–2 nm (Figure 4). This trend was similar in the G12C, G12A, and Q61H simulations, although the rates of change in density differed between the three and the wildtype simulation. All three and the wildtype stabilized between 4 and 6 ns. G12D, G12V, G12S, and G13D all had more significant density fluctuation throughout the simulation. Additionally, when those four mutants were interacting with WT-*HRAS*, WT-*HRAS* increased in density as well. In the G12D mutant, however, they equilibrated between 4 and 6 ns, although WT-*HRAS* is more compact than that in wildtype. G12R notably had a large fluctuation of decreased density between 4 and 6 ns before equilibrating.

##### 3.2.2.2 NRAS

**FIGURE 5:**
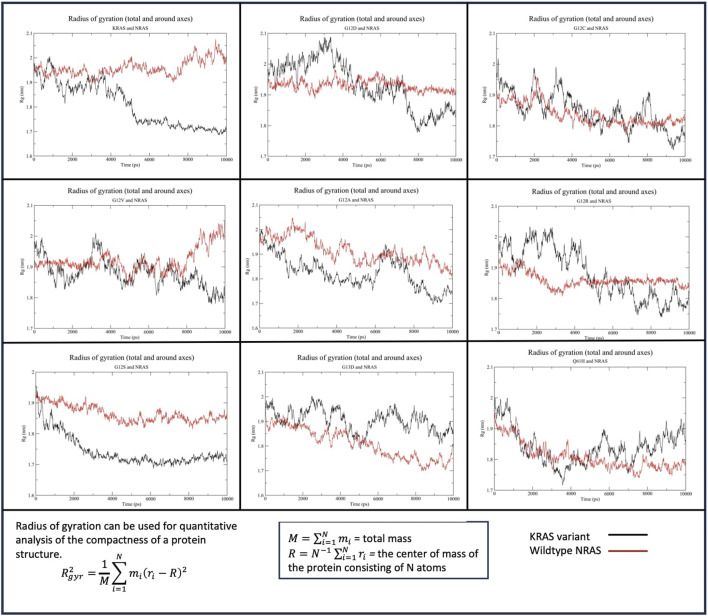
Radius of gyration (Rg) of the *KRAS* variants with WT-*NRAS*. The average distance protein residues from the center of the protein were plotted against time for the *KRAS* variants and WT-*NRAS*. A smaller Rg signified a denser protein, while a higher Rg indicated a decrease in overall compactness. Mutation in *KRAS* was observed to influence the density of both proteins.

The Rg of WT-*KRAS* interacting with WT-*NRAS* indicated that WT-*KRAS* remained at a stable density until 4 ns, when it quickly increased in density before equilibrating at ∼1.7 nm from 5 ns until the end of the simulation (Figure 5). WT-*NRAS* remained at a more consistent density of 1.9–2 nm throughout the simulation. No mutant presented a significantly similar trend with the wildtype. G12S displayed a consistently higher density for the mutant than WT-*NRAS*. G12A similarly had a higher density than WT-*NRAS*, except for a fluctuation of decreased compactness from 6 to 8 ns when the mutant had a similar density to WT-*NRAS*. Interestingly, G13D presented a higher density than WT-*NRAS* in their interaction, which both increased throughout the simulation. G12D and G12R both displayed lower density at the beginning of the simulations than at the ends when they equilibrated. WT-*NRAS* remained at a consistent density when interacting with both of those mutants. G12C and WT-*NRAS* each had a large fluctuation in density as each experienced a gradual increase in density. Q61H underwent a more rapid rate of density increase for the mutant and WT-*NRAS* before the latter equilibrated between 4 and 6 ns. Interestingly, the mutant decreased in density once WT-*NRAS* was equilibrated. Most noteworthy was the Rg of G12V and WT-*NRAS*. The mutant originally had a large fluctuation in density, which decreased significantly at 3 ns. Meanwhile, the WT-*NRAS* originally had a constant equilibrated density until 3 ns when small fluctuations began, and the density decreased to similar levels as WT-*NRAS* in the wildtype simulation.

### 3.3 Quantifying residue–complex interaction energies (per-residue)

#### 3.3.1 HRAS

The total complex line plot of each simulation graphs the overall energetic stability of complex interaction. A negative energy value was expected for all simulations, indicating favorable binding between the proteins. The greater the absolute value of total complex energy indicates the more energy involved in the complex. A low standard deviation from the mean signified the energetic stability of the complex. The average of the total complex energy across all frames of the simulation was calculated as the mean complex energy of each complex. WT-*KRAS* and WT-*HRAS* had a mean complex energy of −2,998.51 kcal/mol and a standard deviation of 19.60 kcal/mol. All variants maintained a similar level of fluctuation in the total complex energy, signifying the energetic stability of the simulations (Table 2). G12C and G12V were the most similar in total energy to the wildtype complex. G12R had the greatest increase in complex energy. All complexes had similar deviations of approximately 20 kcal/mol, indicating consistent stability.

**TABLE 2:** Total complex interaction energy of *KRAS* variants with *HRAS*.

	Wildtype	G12D	G12C	G12V	G12A	G12R	G12S	G13D	Q61H
Mean	−2,998.51	−3,257.23	−2,985.48	−2,984.69	−3,190.69	−3,503.22	−3,283.33	−3,094.03	−3,179.25
STDEV	19.60	23.04	19.84	19.94	21.81	22.28	24.76	20.34	21.46
Standardized mean	__	−258.72	13.03	13.82	−192.18	−504.71	−284.82	−95.52	−180.73

The total complex interaction indicated the overall bound energy of the complex before subtracting the unbound energy (represented in the delta values). Total complex line plot mean, standard deviation, and standardized mean with WT-*KRAS* are listed. The standardized mean is equivalent to the delta of the *KRAS* variants with WT-*KRAS*; the more negative the mean energy value, the higher stability of the complex between the *KRAS* variant and WT-*HRAS*.

The delta line plot of each simulation graphs the overall fluctuation in binding strength between the *KRAS* variant and *HRAS*. A negative binding energy indicated a thermodynamically favored interaction. The average of the delta energy across all frames of the simulation was calculated as the mean binding energy of each complex. Wildtype *KRAS* had a mean binding energy of −46.68 kcal/mol with *HRAS*. The binding of wildtype *KRAS* with *HRAS* maintained the most stable binding strength of all the variants, with a standard deviation of 3.85 kcal/mol. The means were also standardized with the wildtype *KRAS* mean, with a negative value indicating stronger binding than the wildtype. G12D notably was the only variant with a weaker binding energy than WT-*KRAS*, with a positive standardized mean of 1.20 kcal/mol. Additionally, it had a statistically significant greater degree of fluctuation in binding strength. Only G12C had a statistically insignificant *p*-value of 0.1373. All other variants had significant *p*-values with WT-*KRAS*. G13D notably had the greatest binding strength and binding strength fluctuation of all variants. G13D had the most significant standardized mean of −22.04 kcal/mol. Q61H had the second largest negative standardized mean. Of the residue variants mutated at the G12 position, G12S had the greatest standardized mean (Table 3).

**TABLE 3:** Binding strength of *KRAS* variants with *HRAS*.

	Wildtype	G12D	G12C	G12V	G12A	G12R	G12S	G13D	Q61H
Mean	−46.68	−45.48	−46.98	−49.23	−47.21	−51.58	−55.39	−68.72	−60.33
STDEV	3.85	7.64	5.09	5.14	6.44	5.67	5.55	7.66	7.23
Standardized mean	__	1.20	−0.30	−2.55	−0.53	−4.90	−8.71	−22.04	−13.65

Delta line plot mean, standard deviation, and standardized mean with WT-*KRAS* are listed. The standardized mean is equivalent to the delta of the *KRAS* variants with WT-*KRAS*; the more negative the mean energy value, the stronger binding occurs between the *KRAS* variant and *HRAS*. WT-*KRAS* had a mean binding energy of −46.68 kcal/mol. A larger standard deviation indicates a greater fluctuation in binding strength. For the standard mean, a negative value indicates stronger binding between the *KRAS* variant and WT-*HRAS* than WT-*KRAS* and WT-*HRAS*.

#### 3.3.2 NRAS

A low standard deviation from the mean signified the energetic stability of the complex. The WT-*KRAS* and WT-*NRAS* complex had a mean complex energy of −3,418.02 kcal/mol and standard deviation of 20.84 kcal/mol (Table 4). All variants maintained a similar level of fluctuation in the total complex energy, signifying the energetic stability of the simulations. G12V, G13D, and Q61H were the most similar in total energy to the wildtype complex. Although there was much less difference in complex interaction energy overall amongst the *KRAS* variants with WT-*NRAS* compared to *KRAS* variants with WT-*HRAS*, G12D had the greatest decrease in complex energy. G12S was the only mutant complex with more energy than the wildtype. All complexes had similar deviations of approximately 20 kcal/mol, indicating consistent stability.

**TABLE 4:** Total complex interaction energy of *KRAS* variants with *NRAS*.

	Wildtype	G12D	G12C	G12V	G12A	G12R	G12S	G13D	Q61H
Mean	−3,418.02	−3,267.40	−3,301.72	−3,367.86	−3,277.64	−3,340.42	−3,587.86	−3,386.78	−3,455.33
STDEV	20.84	22.64	21.87	21.08	23.99	22.92	23.99	20.37	19.69
Standardized mean	___	150.62	116.29	50.16	140.37	77.59	−169.85	31.24	−37.32

The total complex interaction indicated the overall bound energy of the complex before subtracting the unbound energy (represented in the delta values). Total complex line plot mean, standard deviation, and standardized mean with WT-*KRAS* are listed. The standardized mean is equivalent to the delta of the *KRAS* variants with WT-*KRAS*; the more negative the mean energy value, the higher stability of the complex between the *KRAS* variant and WT-*NRAS*.

WT-*KRAS* had a mean binding energy of −65.66 kcal/mol with WT-*NRAS*, which was statistically significant (>0.05) with greater binding strength than all of the variants except G12V with a mean binding energy of −69.65 (Table 5). Both had relatively high standard deviations. The means were also standardized with the WT-*KRAS* mean, with a negative value indicating stronger binding than the wildtype. G12R and G12S had the least decrease in binding strength with WT-*NRAS*, although G12R had the highest standard deviation of 10.38 kcal/mol.

**TABLE 5:** Binding strength of *KRAS* variants with *NRAS*.

	Wildtype	G12D	G12C	G12V	G12A	G12R	G12S	G13D	Q61H
Mean	−65.66	−45.30	−46.37	−69.65	−42.16	−59.81	−59.19	−50.01	−40.45
STDEV	7.23	6.38	4.07	8.11	6.01	10.38	8.13	4.32	3.93
Standardized mean	__	20.36	19.29	−3.99	23.50	5.85	6.47	15.65	25.22

Delta line plot mean, standard deviation, and standardized mean with wildtype *KRAS* are listed. The standardized mean is equivalent to the delta of the *KRAS* variants with wildtype *KRAS*; the more negative the mean energy value, the stronger binding occurs between the *KRAS* variant and WT-*NRAS*. WT-*KRAS* had a mean binding energy of −65.66 kcal/mol. A larger standard deviation indicates greater fluctuation in binding strength. For the standard mean, a negative value indicates stronger binding between the *KRAS* variant and WT-*NRAS* than WT-*KRAS* and WT-*NRAS*.

### 3.4 Quantifying residue–residue interaction energies (per-wise)

#### 3.4.1 HRAS

Significant residue–residue interactions in each mutant simulation were compared with the residue–residue interactions in the wildtype simulation. Delta energies were calculated for the residues involved in the mutants that were consistent with the wildtype, and then, the average of all simulation values was calculated (Table 6). A larger negative delta energy indicated that there was stronger binding between the residues in the mutant than the wildtype (blue). A larger positive delta energy indicated that there was stronger binding between the residues in the wildtype than the mutant (red). Some residues that were present in the wildtype were not present in the mutant, therefore indicating a stronger energy in the wildtype. The table is inverted diagonally across the center as each pair of residue’s interactions was calculated twice, once for the first residue and again for the second residue. The interaction values were significantly similar between the two calculations. Residues of protein A (WT-*HRAS*) and protein B (*KRAS*) are expected to also interact with other residues of their respective proteins, but of most interest are the pairs of residues between protein A (WT-*HRAS*) and protein B (*KRAS*). However, it is noteworthy that the Gln25 residue in *KRAS* exhibited the strongest interaction with itself. Interestingly, Gln25 was the site of the greatest energy difference, both strong and weak, between mutant and wildtype interactions with WT-*HRAS*.

**TABLE 6:** Average delta per-wise binding energy between *KRAS* and *HRAS*.

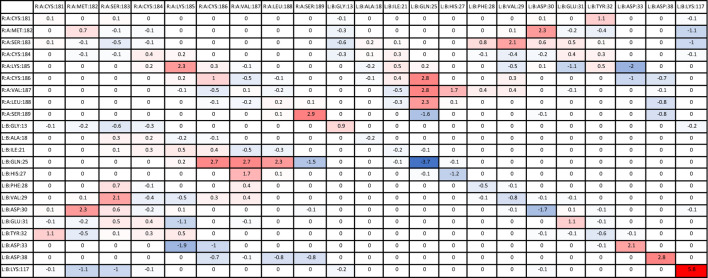

There were additional residues that were not present in the wildtype interaction, but present in mutant interactions, that provided a significant contribution to the binding energy between mutants and WT-*HRAS* (Supplementary Table S1). Only residues that had significant interaction energies (<−1.00 kcal/mol) are listed with those interactions in parentheses. If multiple residues interact with the residue, they are listed chronologically. Most energy values were between −1.00 and −2.00 kcal/mol. No positive interaction energies were observed. Many of these residues were specific to certain mutants, although the significant interactions listed in Table 6 share several similarities between mutants. Specifically, Gly15, Ser17, Pro34, Thr35, Ile36, and Tyr40 in the mutants were commonly involved. Of note, only G12D and G12R had their mutated residues involved in interactions. However, Asp12 did have an interaction value greater than −1.00 kcal/mol. Most notable was that Arg12 had a significant negative interaction involving −14.2 kcal/mol with Glu176 in WT-*HRAS*.

#### 3.4.2 NRAS

The average delta energies were calculated for *KRAS* and WT-*NRAS* interactions similar to WT-*HRAS* (Table 7). More residues were involved between them than between WT-*KRAS* and WT-*HRAS*. The two rounds of energy calculations for each residue pair again confirmed significantly similar values. Notably, Met189 in WT-*NRAS* was involved with the mutant’s strongest and weakest binding strengths compared to the wildtype, although the strongest interaction was with itself. The weakest interaction compared to the wildtype was between Met189 and Thr87 in *KRAS*. Several other residue pairs exhibited weaker binding strength between protein A (WT-*NRAS*) and protein B (*KRAS*) in the mutant than the *KRAS*.

**TABLE 7:** Average delta per-wise binding energy between *KRAS* and *NRAS*.

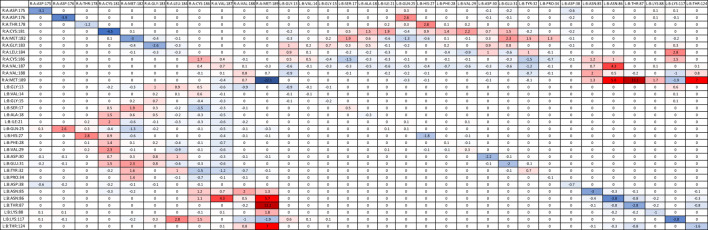

Similar to the simulations with WT-*HRAS*, there were also additional residues that were not present in the wildtype interaction but present in mutant interactions with WT-*NRAS* (Supplementary Table S2). Only residues that had significant interaction energies (<−1.00 kcal/mol) are listed with those interactions in parentheses. If multiple residues interact with the residue, they are listed chronologically. Most energy values were between −1.00 and −2.00 kcal/mol. No positive interaction energies were observed. Again, several residues exhibiting significant interactions were common across the mutants, most notably Asp33 and Tyr40 in the mutant. Notably, Arg12 presented itself to be involved in the interaction in G12R’s simulation, interacting with Cys186, Val187, Val188, and Met189 with energies of −2.62, −3.21, −1.5, and −3.55 kcal/mol, respectively. This was much lower than the amount of energy used in the interaction of Arg12 in the reaction with WT-*HRAS*. Asp12 again appeared to have an interaction value greater than −1.00 kcal/mol. Cys12 was involved in the G12C complex interaction with Val187 and Val188 with energies of −1.24 and −2.81 kcal/mol, respectively. His61 was involved in the Q61H complex interaction with Cys181 with an interaction energy of −1.12 kcal/mol. Interestingly, G12R had an interaction with its wildtype Glu61 residue, unlike any other mutant.

## 4 Discussion

The RAS oncogene family has been studied extensively in terms of protein expression levels and their effects on cellular mechanisms. Our data expand the framework of what has been discovered about the binding patterns of the different *KRAS* mutants with their familial isoforms. Their synergistic behavior is a component discovered in numerous cancer types in different proportions. Therefore, our first goal was to develop a blueprint of the precise structural modifications and binding kinetics interactions. In the process, we illustrated and took note of the similarities and differences between the mutants and the wildtype interactions. Future inhibitory drug design can hopefully utilize these parameters for targeting the isoform interactions as a possible method of reducing oncogenic effects on the cell.

### 4.1 The P-loop and switch I and II regions are significant points of binding for RAS isoforms

**FIGURE 6:**
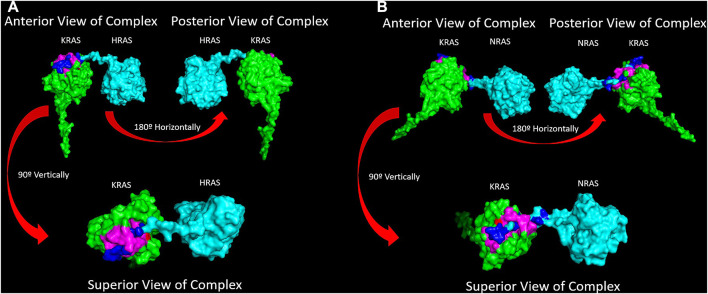
**(A)** A surface texture model of the WT-*KRAS*/WT-*HRAS* is presented. The complex was docked together in the most energetically favorably conformation. The c-terminus tail of WT-*HRAS* was determined to be the most energetically stable interaction point to WT-*KRAS* oriented with its c-terminus tail posteriorly. The residues of the greatest interaction of WT-*KRAS* and WT-*HRAS* are highlighted in pink and dark blue, respectively. The G12 residue is highlighted in red. It was the most common residue mutation analyzed in this study. **(B)** A surface texture model of the WT-*KRAS*/WT-*NRAS* is presented. The complex was docked and simulated in the same manner. Interestingly, opposite to the docking with WT-*HRAS*, the most energetically favorable interaction between the proteins was determined to be the c-terminus tail of WT-*NRAS* bound to WT-*KRAS* with its c-terminus tail oriented anteriorly. It is noteworthy that 5/8 mutants bound in a similar manner, while the G12A, G13D, and Q61H mutants were oriented with their tails posteriorly when bound to WT-*NRAS*, more similar to the WT-*HRAS* interactions. A distinct feature of the WT-*KRAS*/WT-*NRAS* complex is the c-terminus of WT-*KRAS* pointing away from WT-*NRAS*. All mutant *KRAS* proteins pointed parallel to WT-*NRAS*, similar to the WT-*KRAS*/WT-*HRAS* complex.

**FIGURE 7:**
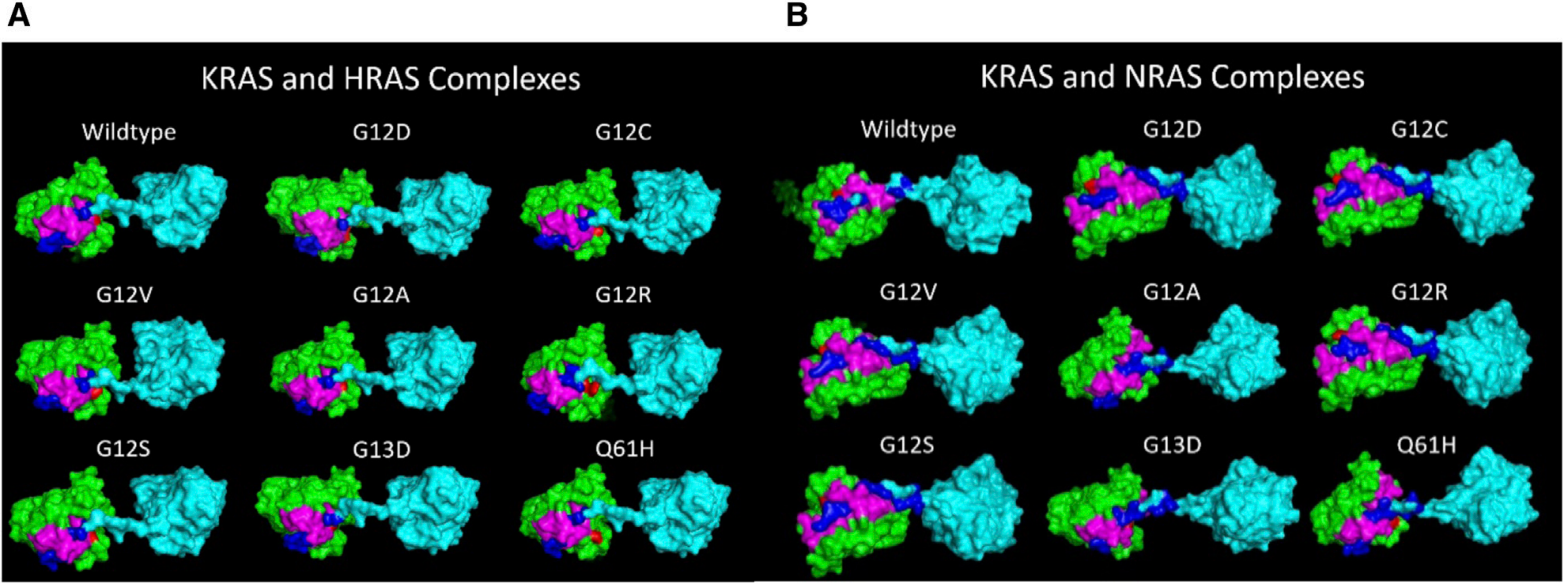
**(A)** Superior views of all the surface texture *KRAS*/WT-*HRAS* complexes are compared here. The tertiary structure of the proteins varies to a degree due to the mutations. The tertiary structure of WT-*HRAS* amongst the complexes also displays differences in orientation. **(B)** Superior views of all the surface texture *KRAS*/WT-*NRAS* complexes are compared here. The G12A, G13D, and Q61H complexes feature the mutant *KRAS* with their c-terminus tails oriented posteriorly. This difference in anterior and posterior positioning of the *KRAS* c-terminus tail impacts the residues and strength of the interaction. Deformity of both proteins in each complex is visible across models.

Through inducing a single mutation into the *KRAS* protein, larger effects on the overall structural and energetic binding trends were significantly notable. Inducing the mutations in *KRAS* initially added torsional strain to the proteins, which impacted the way the most energetically favorable docking conformations with WT-*HRAS* and WT-*NRAS* were calculated in ClusPro. In some cases, these small differences in starting structure demonstrated to have larger impacts on the overall interactions. Our binding kinetic data demonstrate that a majority of the residues with the most significant binding strength between the proteins were located within the p-loop at residues 10–17 and two switch regions (switch I at residues 25–40 and switch II at residues 60–74). The active site where GTP binds and the regions which serve as the binding interfaces for GEF and GAP proteins are structurally and kinetically modified. Therefore, it is supported that the alteration from regular wildtype interactions between *KRAS* and WT-*HRAS* or WT-*NRAS* influences their overall function. There are notable differences between WT-*HRAS* and WT-*NRAS* binding to WT-*KRAS* (Figures 6, 7). The orientation *KRAS* is rotated 180° in relative comparison of the complexes. Furthermore, the tertiary structures of *KRAS* and the isoforms in the complexes exhibit variation.

The 180° rotation difference between the isoform complexes directly correlated with the regions of *KRAS* affected by isoform interactions. The switch II region was active in binding with WT-*KRAS* and WT-*NRAS* in addition to the switch I and p-loop regions, while only the latter two were active in *KRAS-HRAS* complexes. However, most mutations resulted in loss of interactions at the switch II region. In the WT-*KRAS*, G12V, G12R, and G12S complexes with WT-*NRAS*, there was a significantly increased binding strength between proteins (Table 5) compared to the binding strength of *KRAS* with WT-*HRAS*. In contrast, the G12A, G13D, and Q61H complexes with WT-*NRAS*, which did not present the 180° rotation, did not exhibit this increase in binding strength. On the contrary, when G13D and Q61H were bound with WT-*HRAS*, they displayed increased binding strength, without the rotation. Interestingly, although the G12D and G12C mutants presented the 180° rotation, their binding strength remained consistent with mutants that did not. Overall, per-wise energy results indicated that WT-*NRAS* functioned as a stronger inhibitor for types of mutant *KRAS* when 180° rotation is present. Therefore, the development of a pan-*KRAS* mutant inhibitor of those mutants would better be suited to use a small-molecule model, mimicking the structure of the WT-*NRAS* hypervariable region. Additionally, per-wise results indicated the G13D and Q61H mutants are better suited to be inhibited by a small-molecule model mimicking the structure of the WT-*HRAS* hypervariable region.

Previous studies of RAS dimerization identify several interfaces as interaction candidates. A molecular dynamics simulations study exploring the interface site in *KRAS–KRAS* dimers generated a heatmap, indicating the frequency of all *KRAS* residues involved in the many dimer pairings tested. The overall probability of each residue participating was also calculated and represented in a bar chart (Ngo and Garcia, 2022). Consistent with our findings, it was reported that there was substantial involvement of residues within the switch regions and more minimal but still significant involvement of p-loop residues. Notably, they discovered several interfaces involving the negatively charged residues in the switch regions and the hypervariable region of the other *KRAS*, indicating an impact on switch function by the hypervariable region, which presents a similar model to the isoform interactions observed in this study. The identical chemical moieties of the *HRAS* and *NRAS* G-domains are reasonable but not absolutely indicative of similar results in *KRAS*-*HRAS* and *KRAS*-*NRAS* dimers. Fewer small-molecule inhibition studies have been dedicated in exploring the potential of this interface than other interface candidates for RAS dimerization and interactions, such as the ɑ4–ɑ5 helices' interface site, which was not observed in our results (Spencer-Smith et al., 2019; Tang et al., 2023). Thus, our results support increased investigation of the switch region and hypervariable region interface.

In RMSF analysis of the *KRAS* mutants with WT-*HRAS* and WT-*NRAS*, the most significantly affected region was the hypervariable region. The hypervariable regions of WT-*HRAS* and WT-*NRAS* were their main sites of interaction with the *KRAS* mutants, represented by their high mobility, although the amount of mobility varied with the mutant. The hypervariable region of *KRAS* was also observed to range in mobility in RMSF data. The flexibility of the hypervariable region has been noted to serve a number of functions, including serving as a site for post-translational modifications, intracellular transport, anchoring to the membrane, and, of most relevance, regulation through a role in isoform-specific protein–protein interactions and signaling (Abdelkarim et al., 2019; Nussinov et al., 2021). The resulting effect of the altered *KRAS* hypervariable region mobility due to the isoform interaction may impact its functions listed. This is supported by a previous molecular dynamics simulation study, which observed the *KRAS* hypervariable region to function as a possible mediator of *KRAS–KRAS* dimerization. Thus, the conformational changes observed in this study present evidence for impacting *KRAS–KRAS* dimerization, responsible for driving tumorigenic effects.

The GTP hydrolysis-binding site in *KRAS–KRAS* dimers predicted by the aforementioned molecular dynamics simulations study also demonstrates some consistency with our RMSF and per-wise analyses. The study identified the most probable cationic residues involved in GTP hydrolysis by analyzing the interaction between one *KRAS* protomer and the other already bound to a GTP molecule. Of the several cationic interfaces they observed to exist (cationic residues within 5 Å of GTP), most notably, they determined the hypervariable region of *KRAS* to form an interface with GTP for a significant period of time. Hypermobility of the KRAS hypervariable region, induced by an isoform interaction, would impact such interactions. Additionally, they determined the K88 residue to spend the longest period of time forming an interface with GTP, which was observed to interact with WT-*NRAS* in our per-wise analysis. Therefore, there is some indication that GTP hydrolysis is impacted by isoform interactions. Evidence of the switch region and hypervariable region interface being a possible point of inhibition that disrupts *KRAS* dimerization and GTP hydrolysis disarms the previous notion of RAS being “undruggable” due to the high affinity for GTP and the substrate’s large abundance in the cell.

### 4.2 Development of *KRAS* mutant-specific and pan-*KRAS* inhibitors

The development of *KRAS* mutant-specific and pan-*KRAS* inhibitors can take different approaches utilizing the structural and kinetic binding results. Already noted previously, the p-loop and switch regions are presumed to be significant sites of inhibition using small molecules designed to emulate the isoform hypervariable regions. Expanding on this finding, we determined that *KRAS* mutants that were discovered to experience the 180° rotation when bound with WT-*NRAS* would be best inhibited by a small molecule mimicking the WT-*NRAS* hypervariable region. Additional derivations may be concluded from a comparative analysis of the mutants.

In RMSF, Rg, and per-residue analyses, general trends were observed and compared with wildtype interactions (Table 8). There was no mutant interaction with WT-*HRAS* or WT-*NRAS* that was completely structurally or kinetically consistent with the wildtype interaction, although the G12C mutant displayed significant similarities when interacting with WT-*HRAS.* In addition, the G13D mutant displayed significant similarities in RMSF data with the wildtype complexes for each isoform. Despite some mutants resembling similar trends as the wildtype complexes, none was exactly the same and no two complexes were alike each other structurally and kinetically. Interestingly, it was rarely true that the mutant exhibited similar trends during the interaction of WT-*HRAS* vs. with WT-*NRAS*. Structural analysis revealed that G13D was the most similar to the wildtype interaction with WT-*HRAS* and WT-*NRAS*, although only the latter had similar energy values as well. Only G12C presented similar RMSF and Rg trends to the wildtype simulation when interacting with WT-*HRAS*. In all RMSF graphs of *KRAS* mutants with WT-*NRAS*, except G12A, G12V, and G13D, there was a significant decrease in the hypervariable region mobility compared to the wildtype complex, whereas the *KRAS* hypervariable region mobility was less variable when interacting with WT-*HRAS*. Although the overall level of the hypervariable region mobility of *KRAS* mutants interacting with WT-*HRAS* remained similar to the wildtype complex, there are still significant distinctions in slope caused by some hypervariable region residues fluctuating inconsistently compared to the wildtype complex. Aforementioned, the impact of isoform interaction on the *KRAS* hypervariable region presents itself as a mechanism of suppressing its tumorigenic effects.

**TABLE 8:** Overall complex structural and kinetic analysis.

Complex	KRAS variant	Root mean square fluctuation (RMSF)		Radius of gyration (Rg)	
		*KRAS* fluctuation	*HRAS/NRAS* fluctuation	*KRAS* density	*HRAS/NRAS* density
KRAS-HRAS	WT	Standard		Standard	
	G12D	Similar to Standard		Similar to Standard	↑
	G12C	Similar to Standard		Similar to Standard	
	G12V	Similar to Standard	↓	↓	↑
	G12A	↓	Similar to Standard	↓	Similar to Standard
	G12R	Similar to Standard	↓	↓	↑
	G12S	Similar to Standard	↓	↓	↑
	G13D	Similar to Standard		↓	↑
	Q61H	Similar to Standard	↓	Similar to Standard	
KRAS-NRAS	WT	Standard		Standard	
	G12D	↓		↓	↑
	G12C	↓	Similar to Standard	↓	↑
	G12V	Similar to Standard	Similar to Standard	↓	↑
	G12A	Similar to Standard	Similar to Standard	↓	↑
	G12R	↓		↑	
	G12S	↓		↑	
	G13D	Similar to Standard		↓	↑
	Q61H	↓		↓	↑

The overall trends of each *KRAS* variant complex with WT-*HRAS* and WT-*NRAS* were compiled to compare against the respective wildtype complex. Trends similar to the wildtype complex are labeled “Similar to Standard,” while complexes with deviation from the wildtype are indicated to have increased or decreased in specific parameters. Increase and decrease in KRAS and HRAS/NRAS fluctuation refers specifically to the c-termini.

Overall, Rg trends revealed *KRAS* generally became less dense, while the isoform bound to it became denser compared to the wildtype complexes. A small-molecule inhibitor designed with charged and sulfated moieties would similarly disrupt the density of the G-domain region to derive similar suppressive effects of mutant *KRAS*. Thus, it would be valuable to explore, both *in vitro* and *in silico*, the effects of compounds that are similar to the analogs Kobe0065 and Kobe2601, which have both been noted to inhibit *KRAS* and *HRAS* at the switch regions, preventing binding with downstream effectors like *RAF in vitro* (Shima et al., 2013; Keeton et al., 2017). Sulindac sulfide is another frequently studied direct interaction inhibitor of RAS and its mutants at the switch region that decreases downstream signaling of key tumorigenesis pathways like ERK1/2 (Keeton et al., 2017; Rice et al., 2018). The effectiveness of these drugs displays consistency with our results of isoform interactions. Thus, exploring small molecules with similar chemical moieties would be beneficial in the development of pan-*KRAS* inhibitors.

Our kinetic binding data analysis presents the energies of interactions for the residues most involved in the WT-*KRAS*/WT-*HRAS* and WT-*KRAS*/WT-*NRAS* complexes. We calculated the average delta energies of interaction for those residues of all the mutant simulations. Thus, the overall impact on the specific residue binding strengths involved in wildtype interactions was determined. Additionally, we listed the interactions of residues of significant energy of interaction (<−1.00 kcal/mol) that were present in each mutant simulation but not in the wildtype simulation. Thus, a larger effect of the mutations involving additional residues in the complex interaction was determined. When designing inhibitors that are analogous to those described above in this section, it would be beneficial to use *in silico* methods to test interactions with small molecules and the protein complexes for efficient efficacy testing. Additionally, a future investigation on the individual mutation of the most active residues in the protein–protein interactions (such as Gln25 of *KRAS* in the *KRAS*-*HRAS* complexes), studying the effects on the overall complex binding kinetics, would be a novel use of *in silico* techniques. It would allow for specific residue chemical moieties to be singled out as small-molecule inhibition targets and benefit *KRAS* mutant-specific and pan-*KRAS* inhibitors.

### 4.3 Utilizing *KRAS* mutant binding strength to determine the appropriate concentration of RAS inhibitors

Another central goal of this study was to theorize and analyze a possible mechanism for the suppression of oncogenic *KRAS* mutants by WT-*HRAS* and WT-*NRAS* that has already been observed *in vivo* (Tang et al., 2023). A direct interaction between the isoforms is the mechanism explored in this study. The residues involved in the interaction between *KRAS* and its isoforms share commonalities with those involved in *KRAS* dimerization, analyzed in previous studies (Muratcioglu et al., 2015). It can be deduced that direct isoform interactions with *KRAS* may be responsible for the interruption of the dimerization of oncogenic *KRAS*. Our data support previous studies’ hypotheses that altering the proportion of oncogenic *KRAS* and its RAS isoforms may influence the overall oncogenic behavior of *KRAS*. We demonstrate that WT-*KRAS* and its two isoforms have significant and stable binding capabilities, which could serve as a method of inhibition for the mutants if cellular concentrations are adjusted therapeutically. Furthermore, there are notable differences in the interactions between WT-*HRAS* and WT-*NRAS* and the different *KRAS* mutants. The overall effect on each of the *KRAS* mutant proteins when interacting with the two isoforms is described in detail in this study. Certain *KRAS* mutant variants may be more suitable for this inhibition method, as our data exhibited. We propose that the concentration level of the isoforms required to have effective anti-tumorigenic effects on the mutant variants depends on the structural and kinetic characteristics of complex stability and binding strength. Future *in vitro* and *in vivo* experiments with each mutant variant and different intracellular concentrations of the other RAS isoforms would be beneficial in quantifying their effects on *KRAS*-derived tumorigenesis and overall effects on oncogenic *KRAS* actions (dimerization, larger complex formations, signaling, etc.). When testing concentrations of the isoforms, the structural and kinetic characteristics determined in this study may be useful, with mutants that had less stable and lower binding strength with the isoforms requiring higher concentrations than the wildtype and mutants with a higher stability and binding strength (Table 7). Our direct interaction mechanism may be one of several mechanisms occurring. It is also possible that other components of the cell are involved in a complex not explored in this study. Our data may also be beneficial in designing an inhibitory compound with similar effects on the *KRAS* structure as the isoforms do.

## 5 Conclusion

Mutation in the *KRAS* gene is one of the most common oncogenes in many types of cancer. Our study has analyzed a direct interaction approach between the RAS isoforms to develop a blueprint of structural and kinetic components of complexes of the most common *KRAS* mutants and the two isoforms. We have discovered that there are significant differences between the isoform interactions with the different mutants, which could be utilized in inhibitory treatments of mutated *KRAS* in different cancers. Overall, there is a significant interface of interaction between the hypervariable region of WT-*HRAS* or WT-*NRAS* with the G-domain of *KRAS*. Depending on the structural and kinetic strength of the mutants with the isoforms, adjusting cellular concentrations of the isoforms accordingly may effectively serve as a competitive inhibitor of mutated *KRAS* and prevent its oncogenic effects, including dimerization, forming larger complexes, and unregulated signaling. Our paper provides valuable information that can be successfully utilized in developing specific small-molecule inhibitors that will effectively ameliorate the detrimental consequences of *RAS* mutation in different cancer types.

## Data Availability

The datasets presented in this study can be found in online repositories. The names of the repository/repositories and accession number(s) can be found in the article.
