# Supplementary material for: Structural modifications and kinetic effects of KRAS interactions with HRAS and NRAS: an in silico comparative analysis of KRAS mutants
Source: Front Mol Biosci. 2024 Aug 9;11:1436976. doi: 10.3389/fmolb.2024.1436976 (PMC11342451; doi:10.3389/fmolb.2024.1436976)
Supplement: Supplementary file 2 [file Table2.docx]

**Supplementary Table 2. Residues of Significant Interaction Energy Not Present in WT-*KRAS* and WT-*NRAS* Interaction**

| **G12D** | **G12C** | **G12V** | **G12A** | **G12R** | **G12S** | **G13D** | **Q61H** |
| --- | --- | --- | --- | --- | --- | --- | --- |
| — | — | — | — | Ser174 (Gln25) | — | — | — |
| — | — | — | — | Gly177 (Gln25) | Gly177 (Gln43) | Gly177 (Pro121) | — |
| Thr178  (Lys42) | — | — | — | — | Thr178 (Arg41) | — | — |
| — | — | — | — | — | Gln179 (Arg41, Tyr40) | — | — |
| — | — | — | — | — | Gly180 (Tyr40) | — | — |
| **Asp12** | **Cys12 (Val187, Val188)** | **No Mutant** | **No Mutant** | **Arg12 (Cys186, Val187, Val188, Met189)** | **No Mutant** | **No Mutant** | **Gly12 (Cys181, Met182)** |
| Asp33 (Leu184) | Asp33 (Leu184, Pro185, Cys186) | — | — | Asp33 (Leu184, Pro185, Cys186) | Asp33 (Leu184, Pro185) | Asp33 (Cys186, Val187, Val188) | — |
| — | Thr35 (Cys186) | — | — | — | — | — | Thr35 (Cys186) |
| — | — | — | — | — | — | Ile36 (Val188) | — |
| Tyr40 (Gln179, Gyl180) | — | — | Tyr40 (Cys186) | Tyr40 (Cys181, Met182) | Tyr40 (Gln179, Gly180) | — | — |
| — | — | — | — | — | Arg41 (Thr178, Gln179) | — | — |
| Lys42 (Thr178) | — | — | — | — | — | — | — |
| — | — | — | — | — | Gln43 (Gly177) | — | — |
| **No Mutant** | **No Mutant** | **No Mutant** | **No Mutant** | **Gln61 (Cys186)** | **No Mutant** | **No Mutant** | **His61 (Cys181)** |
| — | — | — | — | — | — | Leu120 (Met182) | — |
| — | — | — | — | — | — | Pro121 (Gln179) | — |

The residues of each protein involved in significant interaction (< -1.00 kcal/mol) that were not similar to the residues involved in the WT-*KRAS*/WT-*NRAS* complex are listed. WT-*HRAS* residues are listed first before the mutant residues. Arg12 in the G12R complex was the only mutant that displayed significant interaction at the point of mutation. Although no significant interaction was noted at the mutation site in Q61H, we noted that there was significant interaction at the Gly12 residue, which is the site of the other mutations studied. Asp12 in the G12D complex was observed to have a role in interaction but none exceeded the -1.00 kcal/mol threshold used. The residues Gly177 and Thr178 in WT-NRAS and the residues Asp33, Thr35, and Tyr40 in the *KRAS* mutants all were commonly involved among multiple mutant complexes.
